# Headache and the Eyes

**Published:** 1891-02

**Authors:** 


### HEADACHE AND THE EYES.

Eye strain should be the first thought suggested by any complaint of headache, for it is by far the most common cause of that symptom. The simple existence of headache, therefore, should suggest eye strain, but frequently a careful inquiry as to the manner and time of the attack, and the location of the severest pain, will be almost conclusive as to the origin of the trouble. Often it comes on whenever the eyes are used, and is absent when the eyes have had a proper season of rest.

Congestion, irritability, or inflammation of the eyes and their appendages should always suggest the suspicion of eye strain. A single attack or manifestation of this kind has no special significance, but repeated attacks of inflammation, or prolonged congestion or irritability are suggestive of a continuing cause. A strange thing with reference to eye strain is that it often exists to an exceptional degree without showing any symptoms in the eye. The patient will often say that the eyes are perfectly good and have never caused any irritation.

